# NMR crystallography: structure and properties of materials from solid-state nuclear magnetic resonance observables

**DOI:** 10.1107/S2052252517006042

**Published:** 2017-05-02

**Authors:** David L. Bryce

**Affiliations:** a Department of Chemistry and Biomolecular Sciences and Centre for Catalysis Research and Innovation, University of Ottawa, 10 Marie Curie Private, Ottawa, Ontario K1N 6N5, Canada

**Keywords:** Nuclear magnetic resonance, solid-state NMR, NMR crystallography, dynamics, noncovalent interactions

## Abstract

This topical review provides a brief overview of recent developments in NMR crystallography and related NMR approaches to studying the properties of molecular and ionic solids. Areas of complementarity with diffraction-based methods are underscored. These include the study of disordered systems, of dynamic systems, and other selected examples where NMR can provide unique insights. Highlights from the literature as well as recent work from my own group are discussed.

## Abstract 2

This topical review provides a brief overview of recent developments in NMR crystallography and related NMR approaches to studying the properties of molecular and ionic solids.

## 1. Introduction   

From the early days of nuclear magnetic resonance (NMR), experiments on crystalline samples have provided structural and crystallographic information (Pake, 1948 ▸; Harris *et al.*, 2009 ▸). The information available in a particular case will depend on the nature of the sample, and may range from an internuclear distance to a complete structural model of a complex system such as a protein. In this topical review article, I will provide a short survey of selected recent work in the field of NMR crystallography, a topic on which the IUCr established a Commission in 2014 (*Report of the Executive Committee for 2014*, 2016 ▸), as well as a broader look at various applications of NMR methods to studying the structure and properties of various solids. In this context, NMR methodologies can offer various types of information relevant to the field of crystal engineering (Xu *et al.*, 2016 ▸). NMR crystallographic methods are frequently used in combination with diffraction methods. Long-range order is not a requirement for NMR studies of solids, and so NMR can offer particular advantages for studying disorder, guest dynamics, and amorphous or heterogeneous systems, for example.

The term ‘NMR crystallography’ has been employed in the literature to cover a wide array of approaches to structure determination, refinement, or selection (Harris *et al.*, 2009 ▸; Martineau, 2014 ▸; Martineau *et al.*, 2014 ▸; Xu *et al.*, 2016 ▸). Broadly speaking, these methodologies may be classified into the following categories: (i) *de novo* structure determination using NMR data, (ii) structure refinement against NMR data, and (iii) cross-validation of structural models using NMR data. The first of these is typified by the advanced multidimensional NMR methods used to solve the structures of proteins in the solid state. While such approaches usually rely in part on empirical chemical shift and torsion angle databases, for example, they typically do not need diffraction data or *ab initio* computational methods to produce a structural model. These methods work for proteins in part due to the fact that the primary amino acid sequence is already known, that the basic structures of individual amino acid residues are known, and that the more common secondary structures of proteins are well known and easily identified from NMR data. The above-mentioned advantages of working with proteins may not be present when looking at small organic molecules or inorganic materials, where the most basic structural and bonding information may not be known *a priori*. In this case, one may seek to simultaneously, sequentially, or iteratively incorporate experimental data from many sources, including NMR and diffraction, to produce a structural model which is consistent with all the data. Computational methods, *e.g.* structure optimization by density functional theory (DFT), may also be included (Ashbrook & McKay, 2016 ▸; Beran *et al.*, 2016 ▸). In this category of structure refinement or solution, the data (including NMR data) are used as restraints against which a structural model is refined. In the final category of NMR crystallographic approaches, NMR data (*e.g.* chemical shifts) may be used to select or cross-validate certain structures produced *via* other methods. These other methods may include diffraction refinements and/or advanced computational methods such as crystal structure prediction (CSP) algorithms.

This brief review will provide coverage of recent developments in the above-mentioned areas, with highlights from my own laboratory. Our work has focused on a range of methods and applications associated with crystal structure refinement, as well as providing unique insights into crystallographic symmetry and dynamics in solids. Some of the examples from my laboratory which will be described include (i) a multinuclear magnetic resonance structure refinement protocol based on experimental and computed electric field gradient (EFG) tensors, (ii) insights into noncovalent interactions, such as halogen bonds, *via* combined X-ray and NMR studies, and (iii) novel insights into crystallographic symmetry, as well as molecular dynamics, *via* two-dimensional *J*-resolved NMR experiments.

## 2. Solid-state NMR basics   

Solid-state NMR (SSNMR) spectroscopy provides a nuclear site-specific probe of molecular structure, electronic structure, and overall crystal structure. In comparison with diffraction methods, which benefit to a significant extent from a degree of long-range ordering of molecules in solids, NMR methods tend to provide much more local information. This differentiation arises from the nature of the experiments themselves. The main NMR interactions which give rise to spectral information include magnetic shielding (leading to chemical shifts), indirect nuclear spin–spin coupling (*J*-coupling), and direct dipolar coupling. For nuclei with a spin quantum number (*I*) greater than one-half, the nuclear electric quadrupole interaction also becomes important, and dominates the spectra in many cases. Paramagnetic samples may exhibit additional fine structure and spectral shifts, and the resonances of metallic samples are subject to the Knight shift. The focus here will be largely on diamagnetic samples for which the first four interactions are relevant. This introduction is purposely brief; the interested reader is referred to a variety of excellent monographs for more detailed explanations (Duer, 2005 ▸; Apperley *et al.*, 2012 ▸).

The NMR experiment typically relies on a large external applied magnetic field (*B*_0_) to relieve the degeneracy of the nuclear spin states. The transition associated with the energy gap created by this Zeeman effect has an associated Larmor frequency (ν_0_), 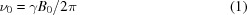
which will depend on the value of *B*_0_ and the identity of the nuclide under investigation (as characterized by its magnetogyric ratio, γ). The Larmor frequency is normally in the range of hundreds of megahertz (MHz) for ^1^H. In real systems, the pure Larmor frequency is modified as a result of the NMR interactions mentioned above. The magnetic shielding interaction, denoted σ, is characterized by an anisotropic and asymmetric second-rank tensor. This interaction effectively modifies the apparent magnetic field at the nucleus, resulting in spectral shifts on the order of parts-per-million (ppm). The NMR experiment does not provide σ values directly, but instead uses chemical shifts (δ) reported relative to a reference compound. Because the magnetic shielding, and thus chemical shift, depends directly and intimately on the electronic and orbital structure at and around the nucleus of interest, these parameters provide distinct spectral signatures for various functional groups, covalent and noncovalent bonding environments, and other structural features.

The chemical shift arises from the interaction of a single nuclear spin with the electrons in a sample. The *J*-coupling and dipolar coupling interactions both arise from the interaction of two nuclear spins. In the case of *J*-coupling, the coupling between the nuclear spins is mediated by the intervening electron spins. The *J*-coupling interaction will result in additional spectral fine structure which is indicative of the nature of the chemical bond between the two atoms comprising the two nuclear spins in question. For example, the different electronic structures associated with single, double, and triple bonds between pairs of C atoms mean that the value of the *J*-coupling between a pair of single, doubly, or triply bonded C atoms will be distinct. The *J*-coupling interaction thus provides another indication of the local molecular and electronic structure. As will be discussed in the following sections, *J*-couplings have also become important indicators of various noncovalent interactions in solution and in the solid state. *J*-coupling between nuclear spins also provides a valuable mechanism for transferring magnetization between nuclear spins in multidimensional NMR experiments, thus enabling correlation spectroscopy which can provide direct atomic connectivity information.

Direct dipolar coupling between a pair, or several, nuclear spins does not involve the electron spins. The dipolar coupling interaction is appealing because, under favorable conditions, it is possible to determine internuclear distances relatively unambiguously. The relationship between the direct dipolar coupling constant and the internuclear distance for a pair of spins is given by: 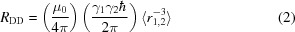
where γ_1_ and γ_2_ are the magnetogyric ratios of the two nuclides involved, and *r*_1,2_ is the distance between them. For a rigid isolated spin pair, the dipolar coupling constant will provide the internuclear distance. The accuracy of such a measurement is compromised when there are molecular dynamics which result in a partial averaging of the dipolar coupling interaction [the angular brackets denote a motional average in equation (2)]. Conversely, if one has independent information on a bond distance (*e.g.* a standard C—H distance in organic or biological samples), then information on the dynamics of this bond vector may be extracted from the measured (reduced) dipolar coupling constant. The most simple determination of bond lengths *via* dipolar couplings only pertains to relatively isolated spin pairs. In contrast, for organic compounds or proteins which contain a large number of dipolar coupled protons, more advanced NMR techniques must be used to properly record and interpret dipolar coupling information.

For quadrupolar nuclei (spin *I* > ½), the nuclear electric quadrupole interaction between the electric field gradient tensor (**V**) at the nucleus and the fixed nuclear electric quadrupole moment (*Q*) results in additional spectral broadening and/or fine structure. As more than 70% of the stable nuclides in the periodic table are quadrupolar, this interaction is of particular importance. Interpretation of the quadrupolar coupling constant (*C*_Q_) and asymmetry parameter (η), obtained from spectral simulations, provides information on the local and long-range symmetry about the nucleus, as well as information on the nature of the bonding to the nucleus and the differentiation between different functional groups, for example.

In solids, all of these interactions are anisotropic, meaning that in a stationary powdered sample, each individual crystallite orientation with respect to the magnetic field will give rise to a different resonance frequency. The resulting spectral ‘powder patterns’ can be quite broad (*e.g.* up to thousands of ppm or more), depending on the nucleus under investigation and the nature of the sample. Spectral resolution may be recovered by a combination of methods, including magic angle spinning (MAS), application of higher magnetic fields in the case of quadrupolar nuclei, double-rotation NMR, multiple quantum MAS, *etc*.

The standard MAS experiment involves rotating a powdered sample packed in a rotor with a diameter on the order of millimeters. The rotor is placed into a probe designed for MAS experiments and spun at an angle of ∼54.74° with respect to the direction of the applied magnetic field. This magic angle is the root of the second-order Legendre polynomial 3*cos* ^2^θ − 1 = 0, a factor which appears in many of the equations which describe the various NMR interactions described above, and which contributes to the spectral broadening giving rise to powder patterns. Spectral resolution is thus increased by rapid MAS because the broadening term disappears. For ^1^H SSNMR, very fast MAS and/or multiple-pulse sequences may be used to suppress the strong dipolar couplings. The precise definitions of ‘fast’ and ‘very fast’ MAS depend on the nature of the interactions to be averaged, but as a general guide, the MAS rate should be comparable to or exceed the magnitude of the broadening. In practice, the typical MAS rates used range from a few kHz for large rotors containing spin-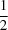 nuclei to over 100 kHz (‘very fast’ or ‘ultra fast’) for quadrupolar nuclei or tightly coupled ^1^H spin systems.

## 3. Applications and examples   

In this section, selected recent examples of NMR crystallographic methods will be presented and described. As mentioned in the *Introduction* (§1), the term ‘NMR crystallography’ encompasses a wide range of approaches to providing structural and crystallographic information on solids. An inspection of the recent literature provides an overview of the various approaches which are currently being developed and applied. Recent special issues of *Solid State Nuclear Magnetic Resonance* (Mafra, 2015 ▸) and *Acta Crystallographica Section C* (Bryce & Taulelle, 2017 ▸) provide a timely overview of the diversity of such methods and applications, and the reader is encouraged to consult these collections.

**Figure 1:**
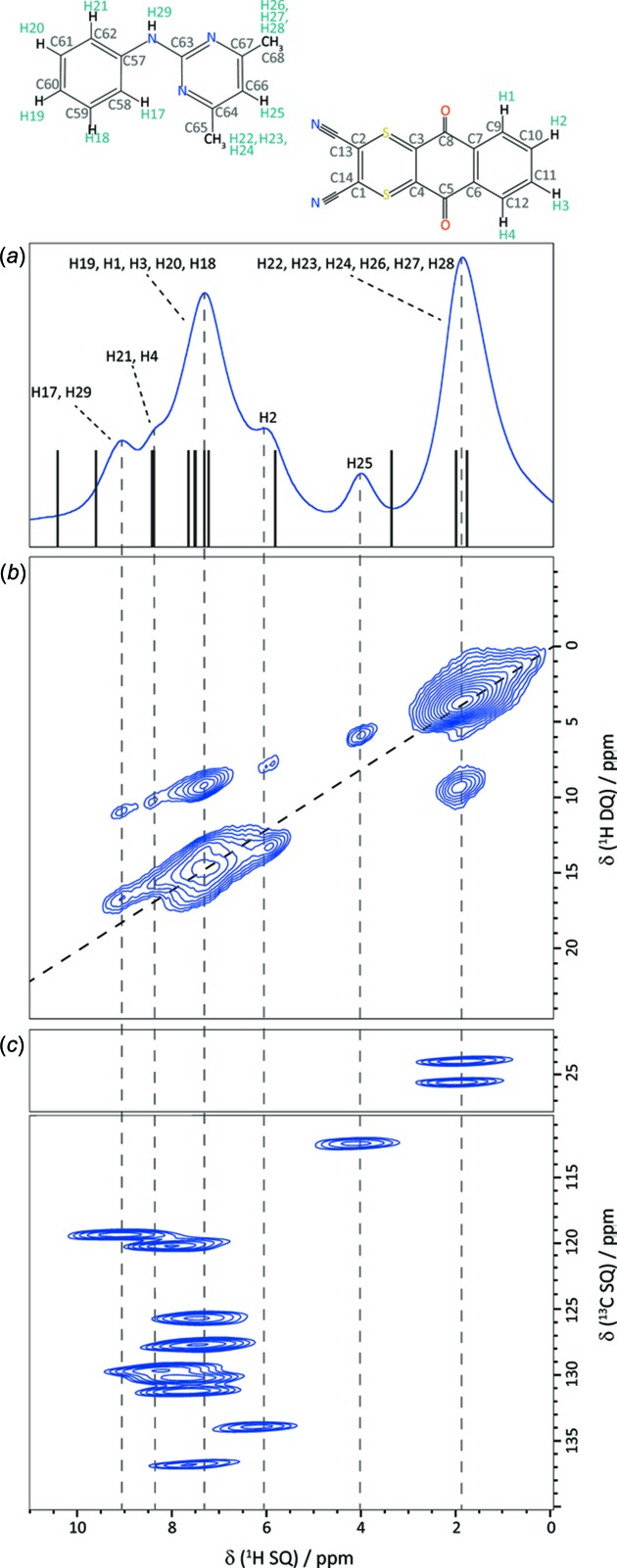
MAS NMR spectra of the di­thia­non–pyrimethanil cocrystal, showing (*a*) a ^1^H MAS one-pulse spectrum, (*b*) a two-dimensional ^1^H double-quantum MAS spectrum and (*c*) a ^1^H–^13^C HETCOR MAS spectrum. The vertical lines in part (*a*) correspond to calculated ^1^H chemical shifts. From Pöppler *et al.* (2017 ▸). Used with permission.

Whereas X-ray diffraction reports on the electron-density distribution, NMR spectroscopy is a probe of the nuclei themselves. Given the ubiquity of hydrogen in naturally occurring and synthetic compounds and biomolecules, NMR approaches can offer some advantages when it comes to looking at hydrogen. While hydrogen only carries one electron, the ^1^H isotope enjoys a 99.6% natural abundance and is among the most spectroscopically receptive nuclides in the periodic table. Given the importance of hydrogen bonding, NMR is particularly useful in this regard. Brown and co-workers have often made particular use of two-dimensional correlation NMR spectra to reveal proton–proton close contacts, as well as carbon–proton proximities, for example. A recent example is that of a 1:1 cocrystal of two fungicides, namely di­thia­non and pyrimethanil (Pöppler *et al.*, 2017 ▸). Shown in Fig. 1 ▸ are examples of the two-dimensional NMR data used to assign resonances and develop a structural model of this cocrystal. In addition to elucidating various close contacts and hydrogen bonds *via* NMR spectroscopy, gauge-including projector-augmented wave (GIPAW) DFT computations were employed in reference to the single-crystal X-ray structure to assess the role of these close contacts in determining the observed chemical shifts.

**Figure 2:**
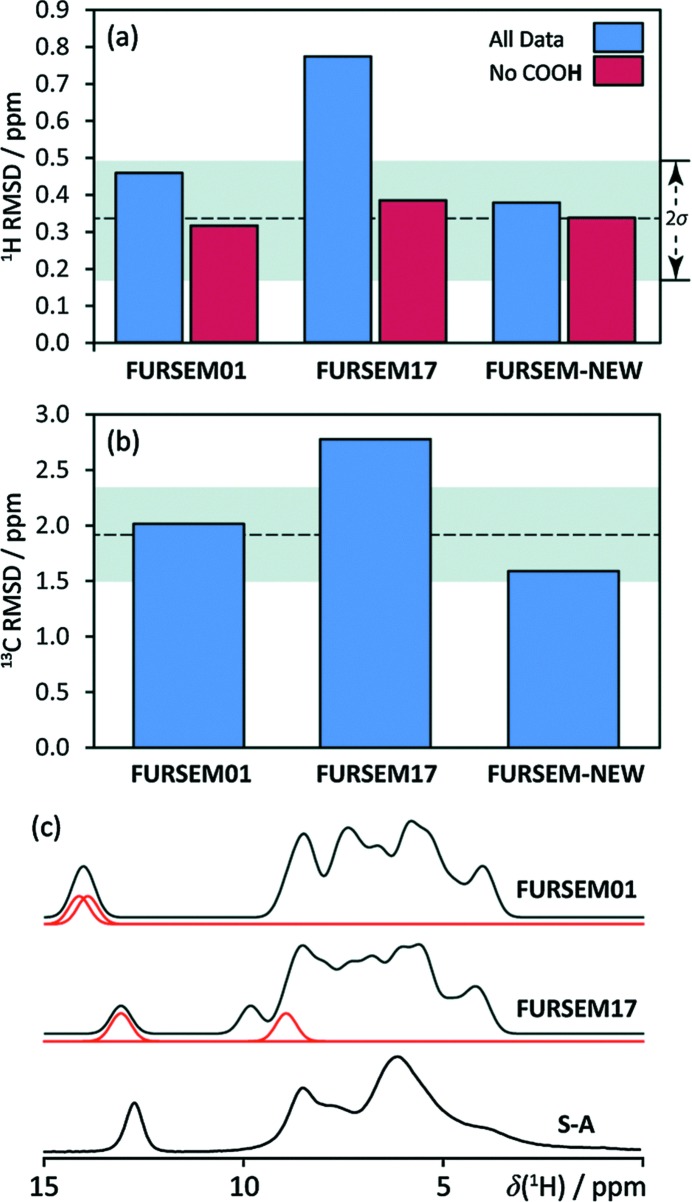
Root-mean-square deviations (RMSD) for isotropic chemical shifts (*a* *) δ*_iso_(^1^H) and (*b*) *δ*_iso_(^13^C) between experimental *δ*_iso_ values for a sample of furosemide from Sigma–Aldrich and a recrystallized sample, with those computed using GIPAW DFT on the H-optimized structures indicated. Grey bands correspond to RMSD ranges established using a series of benchmark organic compounds. The ^1^H RMSD is sensitive to the position of the COOH hydrogen, as shown by RMSD values which include (blue) and do not include (red) this site. (*c*) A comparison of the calculated [CSD refcodes FURSEM01 (Lamotte *et al.*, 1978 ▸) and FURSEM17 (Bolukbasi & Yilmaz, 2012 ▸)] and experimental (from Sigma–Aldrich) ^1^H NMR spectra. Red traces in (*c*) correspond to the carboxyl H atom from each crystallographically unique furosemide molecule. On the basis of the low RMSDs shown in (*a*) and (*b*), the diffraction structures FURSEM01 (and FURSEM-NEW) have been verified by NMR crystallography. From Widdifield *et al.* (2016 ▸). Published by The Royal Society of Chemistry. Used with permission.

The group of Hodgkinson has made several recent contributions to NMR crystallography, including the study of amorphous organic compounds (Skotnicki *et al.*, 2016 ▸), characterizing the role of dynamic water molecules in the drug sildenafil citrate (Abraham *et al.*, 2016 ▸), and testing the limits of their methods in a case study of a 1:2 caffeine–citric acid hydrate cocrystals (Kerr *et al.*, 2016 ▸). In their latest study of a 1:1 cocrystal of naproxen with picolinamide (Kerr *et al.*, 2017 ▸), an NMR crystallography approach was used to study the H-atom positions in the two crystallographically distinct COOH–CONH hydrogen-bonded dimers. Proton SSNMR was employed to distinguish between the two carboxyl protons, in spite of their similar crystallographic environments. A single-crystal X-ray diffraction structure of the cocrystal was refined using DFT, resulting in a final structure in best agreement with the experimentally measured ^1^H and ^13^C chemical shifts. This combination of methods allowed the authors to conclude, on the basis of the final H-atom positions, that the naproxen–picolinamide system is indeed a cocrystal rather than a salt. The importance of accurate H-atom positions, as well as the level of detail which ^1^H SSNMR can provide even for powdered molecular organics, is nicely highlighted in a study of furosemide by Widdifield *et al.* (2016 ▸). Shown in Fig. 2 ▸ are examples of the types of root-mean-square deviations (RMSDs) between experimental and computed chemical shifts used to differentiate between possible structures. Their work demonstrated the value of NMR crystallography in verifying the accuracy of crystal structures found in the Cambridge Structural Database (CSD; Hodgkinson & Widdifield, 2016 ▸; Groom *et al.*, 2016 ▸).

Despite the advantages of using ^1^H SSNMR as a tool for NMR crystallography noted above, motional averaging of interactions due to the small mass of this isotope could add an extra layer of complexity *en route* to highly accurate structural models. For this reason, recent work has focused on the proper treatment of the effects of dynamics on proton chemical shifts, but also on the chemical shift of other isotopes, such as ^13^C and ^15^N, as well as electric field gradients for deuterium (Dračínský *et al.*, 2016 ▸). Specifically, Dračínský *et al.* have explored the temperature dependence of these NMR parameters as determined from path integral molecular dynamics (PIMD) simulations. Their approach convolutes calculated magnetic shielding or EFG tensor components with probability distribution functions of selected bond lengths and angles obtained from DFT/PIMD simulations at various temperatures. They conclude that this method is a particularly universal way to account for dynamical averaging in solids.

Li *et al.* (2017 ▸) have presented a custom-made molecular dynamics force field for NMR crystallography and used it to assess the importance of motion on the selection of the correct structure of cocaine (Baias *et al.*, 2013 ▸). In the work of Baias, it was noted that while ^1^H chemical shifts could be used to identify the correct structure of various small organic molecules produced by CSP methods, ^13^C chemical shifts were not as useful or sensitive in this regard, at least in the cases they studied. Interestingly, the study of Li *et al.* (2017 ▸) concludes that the influence of motional averaging on the ^1^H and ^13^C isotropic chemical shifts is minimal, and that the errors inherent to the GIPAW method are actually the limiting factor in obtaining more accurate values. Hofstetter & Emsley (2017 ▸) have recently reported on the concept of positional variance in NMR crystallography, providing an ‘*ORTEP*’-style image of positional uncertainties derived from NMR. Their approach uses molecular dynamics simulations, DFT calculations, and experimental NMR data, to provide an average positional accuracy for each atom in a crystal structure. In the case of cocaine, they find a positional RMSD of 0.17 Å, which is 2.5 times less than that obtained from a single-crystal X-ray diffraction structure.

**Figure 3:**
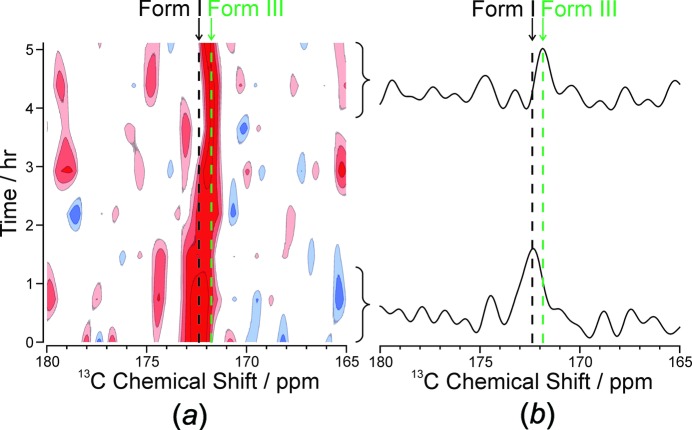
*In situ* ^1^H→^13^C cross polarization NMR spectra recorded as a function of time during the crystallization of *m*-aminobenzoic acid (*m*-ABA) from methanol, showing the region of the spectrum containing the peak for the carboxyl­ate group. The known peak positions for the carboxyl­ate groups in Forms I and III are highlighted by dashed lines. (*a*) The intensity contour plot showing all spectra recorded as a function of time during the *in situ* study. (*b*) Summation of the first two spectra (bottom) and the last two spectra (top) recorded during the *in situ* study. Figure from Harris *et al.* (2017 ▸). Used with permission.

Harris *et al.* (2017 ▸) have been developing various interesting approaches for the *in situ* time-resolved monitoring of crystallization processes. These methods are of particular interest for the discovery of new polymorphs and of metastable phases, somewhat in analogy to the time-resolved diffraction experiments described by Friščić *et al.* (2013 ▸). Harris has described the *in situ* monitoring of polymorphic evolution during crystallization, as well as the discovery of new polymorphs. As an example of the first, polymorphic evolution was noted in the crystallization of *m*-amino­benzoic acid (*m*-ABA) from methanol. Five polymorphs of *m*-ABA are known. Each of these is differentiated by ^13^C SSNMR spectroscopy (Hughes *et al.*, 2014 ▸), which permits the polymorphs present during *in situ* crystallization experiments to be identified. The results of a series of such *in situ* experiments are shown in Fig. 3 ▸, where, in this case, two forms known as Forms I and III are distinguished; the former evolves to give the latter over a period of hours. The CLASSIC NMR experiment (Combined Liquid- and Solid-State *in-situ* Crystallization NMR) com­bines the advantages of SSNMR with those of solution NMR to gain insight into the changes which are occurring in solution during the crystallization process (Hughes *et al.*, 2014 ▸). This experiment can thus elucidate complementary changes that occur in the solid and solution in real time. Harris and co-workers have presented several examples, including an examination of crystallization from a solution of urea, 1,8-di­bromo­octane, and tetra­decane in methanol, where competitive inclusion of the two guest mol­ecules in the urea host tunnel structure was probed during crystal growth (Hughes *et al.*, 2015 ▸).

**Figure 4:**
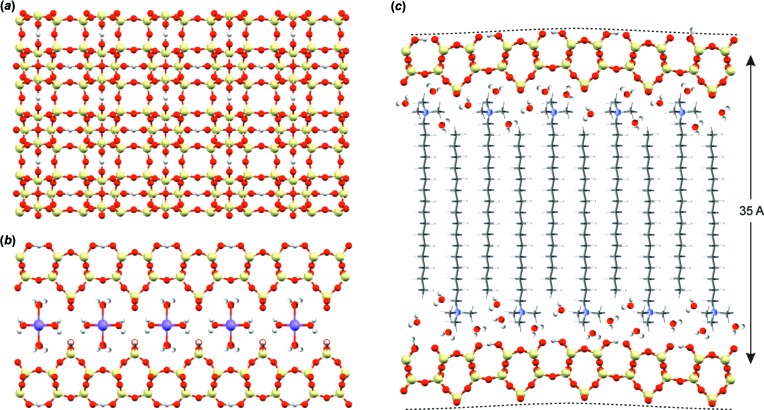
(*a*) The structure of the silicate layer of RUB-18 (Vortmann *et al.*, 1997 ▸), viewed perpendicular to the layer along the *c* axis. (*b*) Two adjacent silicate layers of the RUB-18 structure, with the hydrated sodium ions in the inter­layer space. (*c*) The proposed structure of the C_16_NMe_3_^+^ surfactant-templated layered silicate material based on PXRD and solid-state NMR data. The curved dashed lines illustrate how the silicate layers may be distorted. From Brouwer *et al.* (2017 ▸). Used with permission.

Brouwer and co-workers have presented several advances in NMR crystallographic approaches to characterizing various framework materials, including zeolites, aluminophosphates, and silicate frameworks, some of which pose challenges for diffraction methods because they are only partially ordered (Brouwer, 2008 ▸, 2013 ▸; Brouwer & Horvath, 2015 ▸). An interesting graph theory methodology was employed to solve the structure of zeolite ITQ-4 using PXRD data and a single ^29^Si double-quantum NMR correlation spectrum, for example (Brouwer & Langendoen, 2013 ▸). This group’s most recent work focuses on a partially ordered surfactant-templated layered silicate material (Brouwer *et al.*, 2017 ▸). Such materials can pose challenges for diffraction methods if adjacent layers are not stacked in a regular manner. Thus, the combination of ^29^Si SSNMR, PXRD, and computational chemistry revealed important structural details unavailable from a single class of experiments. In particular, their work showed that the structure of the silicate layer of this layered material templated with cetyltri­methyl­ammonium cations is isostructural with the silicate layer of a previously reported material referred to as ilerite, octosilicate, or RUB-18 (Fig. 4 ▸). Taulelle and co-workers have also made substantial contributions, in particular to the NMR crystallography of layered materials and nanoporous materials (Taulelle *et al.*, 2013 ▸). Two valuable recent examples include the characterization of layered aluminophosphates by synchrotron powder diffraction and NMR crystallography (Bouchevreau *et al.*, 2013*a* ▸) and further related work on such materials (Bouchevreau *et al.*, 2013*b* ▸). Dawson *et al.* (2017*a* ▸) have recently described a modification to the published structure of the aluminophosphate AlPO-53(A) on the basis of NMR crystallographic methods, including DFT modeling of various candidate structures. The hydration of aluminophosphate JDF-2 to AlPO-53(A) was studied *via* multinuclear magnetic resonance, including ^13^C SSNMR spectroscopy of occluded methyl­ammonium cations. The resulting modified AlPO-53(A) structure features re­oriented cations and partially occupied H_2_O sites.

Recent work by Ashbrook and co-workers has nicely demonstrated the power of multinuclear magnetic resonance as applied to characterizing dynamics and disorder in inorganic solids (Moran *et al.*, 2017 ▸). For example, several insights into the phase composition and disorder in La_2_(Sn,Ti)_2_O_7_ ceramics were identified through a combination of ^119^Sn NMR spectroscopy, XRD, and DFT calculations (Fernandes *et al.*, 2016 ▸). In particular, a significant two-phase region in the series was identified which was not predicted based on radius ratio rules. This group’s work on aluminophosphates and on using SSNMR to study dynamics is well exemplified by their recent publication on six forms of AlPO-34 generated from six different structure-directing agents (Dawson *et al.*, 2017*b* ▸). For instance, variable-temperature ^27^Al SSNMR revealed microsecond timescale dynamics in all forms of AlPO-34. Two different motional regimes were observed, depending on whether structural water was present.

Specific distances between quadrupolar and spin-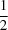 nuclei in a polyoxovanadate cluster were measured using advanced recoupling experiments (DANTE-S-REDOR). Pourpoint *et al.* (2014 ▸) showed how frequency-selective experiments could be used to measure heteronuclear dipolar couplings between ^51^V (spin-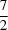) and ^1^H. These and related experiments can be applied to a wide range of related materials which contain quadrupolar nuclei, including zeolites, aluminophosphates, and other polyoxometalates. Analogous distance measurements have been reported by Bonhomme and co-workers in various inorganic materials and nanobuilding blocks (Laurencin *et al.*, 2016 ▸) and imidazolium–silica-based nanoparticle networks (Neouze *et al.*, 2014 ▸).

Leclaire *et al.* (2016 ▸) recently reported what is probably one of the most complex systems, along with proteins, for which SSNMR has been able to provide a complete structure to date. Importantly, in their study, only the molecular formula was available at the outset of the structure solution process. In the case of proteins, the complete primary structure (amino acid sequence) is typically available. Leclaire *et al.* describe the three-dimensional structure of a CO_2_-based organic framework which was obtained from a series of ^1^H, ^13^C, and ^15^N two-dimensional through-bond (*J*-based) and through-space (di­polar) correlation NMR experiments. Molecular-mechanics/quantum-mechanics calculations were used to generate various model structures which were then assessed against the experimental and calculated NMR parameters. Interestingly, PXRD data were not found to be particularly useful in selecting the best final structure, although this is in part due to the failure of the indexing procedure which led to several possible structure solutions, as well as dispersion in some of the chain lengths in the prepared material. This study therefore provides a nice example of where NMR crystallography clearly offers information which is complementary to that available from PXRD studies, and also demonstrates the differing sensitivities of the two methodologies to different aspects of the crystal structure.

Another complex example is that of Caulkins *et al.* (2016 ▸), who employed an integrated combination of multinuclear solid-state magnetic resonance, X-ray diffraction, and com­putational chemistry to characterize a carbanionic intermediate in tryptophan synthase. Their studies established a model of protonation states for ionizable groups on the cofactor, substrates, and proximal catalytic residues. One of the main findings from their study was that a deprotonated pyridine N atom on pyridoxal-5-phosphate precluded the formation of a quinonoid species, and that there is an equilibrium between the phenolic and protonated Schiff base forms of this intermediate. Their work provides a clear example of the value of NMR crystallography in characterizing chemical structure and dynamics within enzyme active sites.

All examples described thus far are of diamagnetic solids. Stebbins and co-workers have reviewed the applications of high-resolution solid-state NMR to the study of silicate, phosphate, and oxide materials with relatively low concentrations of paramagnetic ions, where spectral resolution can remain high enough to distinguish interactions between NMR-observed nuclides and one or more magnetic neighbors in different bonding configurations in the first, second, and even farther cation shells (Stebbins *et al.*, 2017 ▸). This work discusses the value and practical considerations associated with employing paramagnetic effects to gain structural information, in particular concerning short-range order. Applications to a range of inorganic materials are presented, including to pyrochlores, zircon, rare earth cations in xenotime and monazite, transition metal cations in olivine, garnets, and simple cubic oxides. This short review provides a timely introduction to the burgeoning area of NMR crystallography of paramagnetic inorganic samples.

The group of Grey has reported several important advances in the structural and functional characterization of oxides and related energy materials of importance to batteries and supercapacitors (Pecher *et al.*, 2017 ▸; Griffin *et al.*, 2016 ▸). The structural information obtained from SSNMR spectroscopy can provide valuable insights into the mechanisms of operation of such materials. Recent examples include the characterization of the local oxygen environments in paramagnetic battery materials (lithium transition metal oxides) *via* ^17^O NMR and DFT computations (Seymour *et al.*, 2016 ▸). From a structural standpoint, this work also revealed additional ^17^O resonances which were ascribed to stacking faults within the structure of Li_2_MnO_3_. The paramagnetic mixed ionic electronic conductor (MIEC) La_2_NiO_4+δ_ was also probed by ^17^O SSNMR spectroscopy, and three distinct crystallographic O-atom sites were identified (Halat *et al.*, 2016 ▸). Structural distortions among axial O-atom sites, arising from the nonstoichiometric incorporation of interstitial oxygen were resolved and identified by advanced MAS NMR experiments. Spectra acquired at higher temperatures revealed the onset of interstitial oxide motion and exchange with axial sites at approximately 403 K, and this was deemed to be associated with an orthorhombic to tetragonal phase transition. The ability of multinuclear magnetic resonance to identify and characterize structural defects in battery materials has been further demonstrated by the Grey group in related studies (Lee *et al.*, 2017 ▸; Clément *et al.*, 2016 ▸).

**Figure 5:**
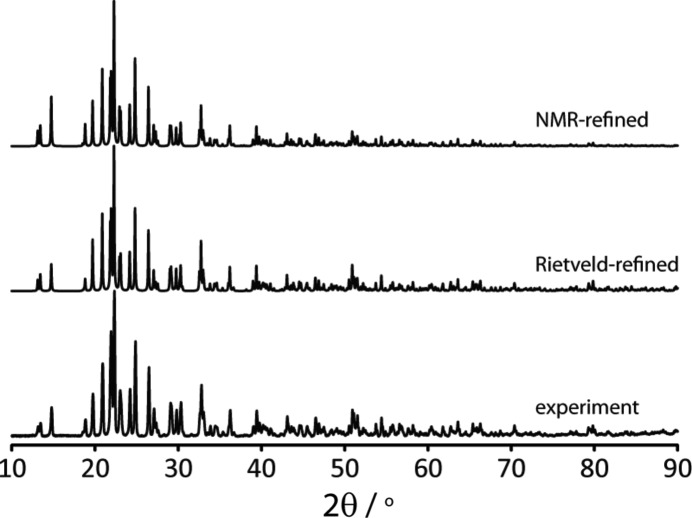
Comparison of powder X-ray diffractograms for ZrMgMo_3_O_12_, a zero thermal-expansion material. Bottom: experimental. Middle: simulation based on a structural model obtained from Rietveld refinement. Top: simulation based on a structural model obtained from NMR crystallography (Romao *et al.*, 2015 ▸). Although there are no significant differences between the traces identifiable by eye, the NMR-refined structure was shown to be in significantly better agreement with the experimental NMR data.

We have developed and applied a multinuclear magnetic resonance crystallographic structure refinement and cross-validation protocol using experimental and computed electric field gradients (Perras & Bryce, 2012 ▸). This approach was first developed and tested on the nonlinear optical material Na_2_Al_2_B_2_O_7_ (NABO). Rather than using NMR data solely as a cross-validation of models derived from X-ray diffraction or DFT optimization, we sought to incorporate the experimental quadrupolar coupling tensors as active restraints in the optimization process. First, high-quality quadrupolar coupling data available from single-crystal NMR studies on model compounds were used to establish correlations with DFT-computed data. Only high-quality structures, typically from neutron diffraction studies, were used in this calibration step. The calibration also provided the value of a coefficient to properly weigh the experimental data *versus* the DFT energy. A series of SSNMR experiments on NABO provided quadrupolar coupling and chemical shift information for ^23^Na, ^17^O, ^27^Al, and ^11^B. Using a model derived from PXRD as a starting point, an iterative structural refinement process was employed to provide a final structure which best simultaneously satisfied all experimental quadrupolar coupling data. The resulting structure was only marginally higher in energy than the pure DFT-optimized structure, but was in far better agreement with the experimental NMR data. The final structure was then also subjected to an independent cross-validation using experimental and computed chemical shifts for ^23^Na, ^17^O, ^27^Al, and ^11^B. The above methodology was extended and applied to ZrMgMo_3_O_12_, a zero thermal-expansion material (Romao *et al.*, 2015 ▸). As also shown for Na_2_Al_2_B_2_O_7_, the resulting structure was only marginally higher in energy than the pure DFT-optimized structure, but was in far better agreement with the experimental ^91^Zr, ^25^Mg, ^95^Mo, and ^17^O SSNMR data. A simple visual inspection of the powder X-ray diffractograms for the initial Rietveld model and final NMR structural models does not show any significant differences (Fig. 5 ▸), yet the agreement with NMR data for the final model is far superior to that obtained before the structure refinement process, as shown by a cost function (Romao *et al.*, 2015 ▸). This demonstrates the particular sensitivity of NMR interactions to the precise positions of atoms in the crystal. Other recent NMR crystallography examples highlight the combined utility of the quadrupolar interaction, chemical shift tensor, and dipolar coupling constant for providing structural insights in metal complexes and sodium diphosphates (Perras *et al.*, 2013 ▸; Widdifield *et al.*, 2015 ▸).

**Figure 6:**
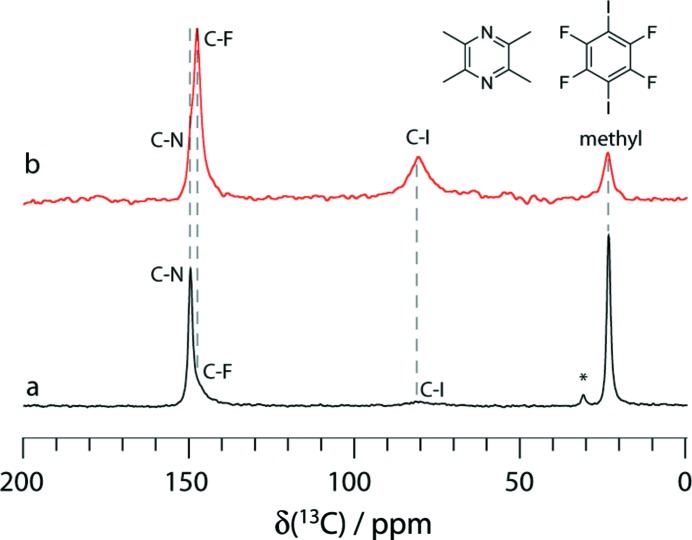
Comparison of the ^13^C CP/MAS spectra of the 1,4-di­iodo­tetra­fluoro­benzene–2,3,5,6-tetra­methyl­pyrazine cocrystal using (*a*) ^1^H cross polarization and (*b*) ^19^F cross polarization. Dashed lines are added as a guide. The labels ‘C-F’ and ‘C-I’ denote the C atom covalently bonded to fluorine and iodine of the halogen-bond donor, respectively. The label ‘C-N’ denotes the C atom covalently bonded to nitro­gen of the halogen-bond acceptor. Cross polarization from the ^1^H in the 2,3,5,6-tetra­methyl­pyrazine moiety reveals weak C—F and C—I resonances, while cross polarization from ^19^F in 1,4-di­iodo­tetra­fluoro­benzene shows peaks due to the C—N and methyl C atoms; these experiments confirm that the two components have cocrystallized. From Szell *et al.* (2017 ▸). Used with permission.

Much of our recent work has focused on the applications of SSNMR spectroscopy to the study of halogen bonds (Szell & Bryce, 2016*a* ▸; Xu *et al.*, 2015 ▸; Viger-Gravel *et al.*, 2014*a* ▸,*b* ▸). A halogen bond exists when there is evidence of a net attractive interaction between an electrophilic region of a halogen atom within a molecule, and an electron-rich moiety (*e.g.* Lewis base, nucleophile) on the same or another molecular entity. Such interactions are highly directional and may be comparable in strength to hydrogen bonds. We have often employed mechanochemical approaches to prepare various halogen-bonded materials and frameworks. Much of this work has been reviewed recently (Bryce & Viger-Gravel, 2015 ▸; Szell & Bryce, 2016*b* ▸; Cerreia Vioglio *et al.*, 2016 ▸) and I briefly describe here only our most recent publication in this area, wherein we reported a ^13^C and ^19^F SSNMR, and X-ray crystallographic study of halogen-bonded frameworks featuring nitro­gen-containing heterocycles (Szell *et al.*, 2017 ▸). In this study, single-crystal XRD, PXRD, high-resolution solid-state NMR, and computational chemistry were used sequentially to elucidate the formation and structural features of the frameworks, rather than in an integrated fashion. In particular, as has been shown previously, resonance shifts in the spectra of several nuclides were shown to be indicative of cocrystal formation. Cross polarization (CP) *via* the dipolar interaction from ^19^F spins on the halogen-bond donors (1,4-di­iodo­tetra­fluoro­benzene and 1,3,5-tri­fluoro-2,4,6-tri­iodo­benzene) to ^13^C spins on the halogen-bond acceptors (*e.g.* acridine, 1,10-phenanthroline, 2,3,5,6-tetra­methyl­pyrazine, and hexa­methyl­ene­tetramine) provided conclusive direct information on co­crystal formation (Fig. 6 ▸). This cross polarization process was interpreted in terms of the ^19^F–^13^C second moments, which are directly reflective of the three-dimensional structures of the cocrystals. Taken together, the XRD/DFT/NMR approach described in this work was shown to provide final structures in best agreement with all experimental data.

**Figure 7:**
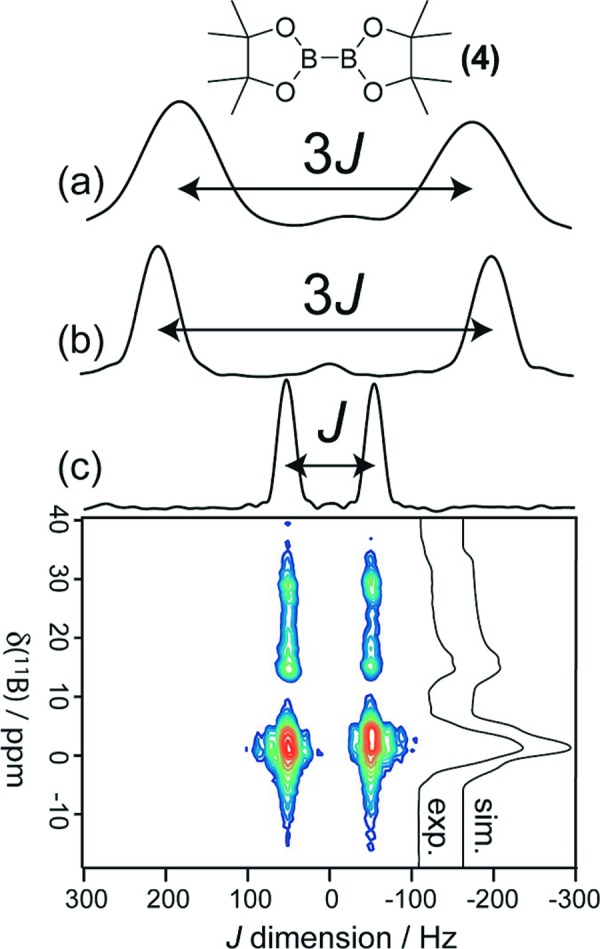
Double-quantum filtered *J*-resolved solid-state NMR spectra for (*a*) bis­(pinacolato)diboron, (*b*) bis­(catecholato)diboron, and (*c*) an N-heterocyclic carbene complexed analogue of bis­(catecholato)diboron. MAS NMR spectra (exp, sim) of the NHC complex are also shown in (*c*). The amplified 3*J* splitting shows that the B atoms are related by a high-symmetry operation, such as an inversion centre, in (*a*) and (*b*), while the symmetry is broken in (*c*). From Perras & Bryce (2013 ▸). Used with permission.

While much of NMR crystallography focuses on molecular structural information, specific details of direct relevance to the space group and the number of molecules in the asymmetric unit are also available under favorable circumstances (Taulelle, 2004 ▸). For example, in an organic molecule, each crystallographically distinct C atom gives rise to a distinct peak in the solid-state ^13^C NMR spectrum (barring fortuitous peak overlap). It is not hard to see how, with some knowledge of the basic molecular formula, a high-resolution ^13^C SSNMR spectrum can tell us about the number of molecules in the asymmetric unit. We have recently developed a series of two-dimensional *J*-resolved SSNMR experiments for pairs of quadrupolar nuclei, the results of which can provide direct insight into the crystallographic symmetry of the system (Perras & Bryce, 2013 ▸, 2015 ▸). Consider for example, a pair of ^11^B spins (*I* = 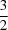) in a molecule such as bis­(cate­chol­ato)diboron. The *J*-resolved MAS NMR experiment will yield in its indirect dimension a doublet, the splitting of which is equal to the value of *J*(^11^B, ^11^B) if the two B atoms are not related by a high-symmetry operation, such as an inversion centre (Fig. 7 ▸). On the other hand, if the two B atoms are related by a crystallographic inversion centre, the doublet splitting is amplified by a factor of (2*I* + 3)(2*I* − 1)/4, which is equal to three for ^11^B. Such experiments have been applied successfully to unambiguously provide information on the space-group symmetry of several compounds featuring quadrupolar spin pairs, including those with boron–boron, manganese–manganese, and gallium–gallium bonds (Perras & Bryce, 2013 ▸, 2014*a* ▸,*b* ▸, 2015 ▸; Kobera *et al.*, 2016 ▸). One may establish independently and unambiguously whether the observed spectral splittings are equal to *J* or whether they are amplified by either (i) calibrating the method on a series of systems with known symmetries, (ii) quantum chemical calculations of the *J*-couplings, and/or (iii) performing analogous *J*-resolved NMR experiments on stationary (rather than spinning) powders and analyzing the sense of the anisotropic powder patterns produced (Perras & Bryce, 2014*b* ▸).

A valuable extension of the above-mentioned *J*-resolved experiments is their application to the study of molecular dynamics in solids (Wong *et al.*, 2017 ▸). We have recently shown how rapid dynamics can render two boron spins effectively magnetically equivalent (related by an inversion centre), even when the single-crystal X-ray structure shows that the two spins are crystallographically distinct at lower temperature. This method for identifying and studying molecular dynamics in solids provides a new tool for differentiating between static and dynamic disorder.

## 4. Concluding remarks   

This brief topical review has focused on selected recent papers in the area of NMR crystallography and related applications of NMR to the study of solids. These fields are vast and the coverage presented here provides only a few snapshots of recent developments. With the support of the IUCr in the form of the Commission on NMR Crystallography, as well as a strong representation for NMR spectroscopy at the 24th Congress and General Assembly of the IUCr in Hyderabad, the future bodes well for increasingly productive synergies between NMR and diffraction-based methods for crystallography. The most translational and productive advances will undoubtedly arise from approaches which play to the strengths of the two methodologies.
